# Plasma miR-155, miR-203, and miR-205 are Biomarkers for Monitoring of Primary Cutaneous T-Cell Lymphomas

**DOI:** 10.3390/ijms18102136

**Published:** 2017-10-15

**Authors:** Nina Dusílková, Petra Bašová, Jindřich Polívka, Ondřej Kodet, Vojtěch Kulvait, Michal Pešta, Marek Trněný, Tomáš Stopka

**Affiliations:** 1 BIOCEV, First Faculty of Medicine, Charles University, 25250 Vestec, Czech Republic; nina.dusilkova@lf1.cuni.cz (N.D.); basova.petra@gmail.com (P.B.); ondrej.kodet@lf1.cuni.cz (O.K.); kulvait@gmail.com (V.K.); 2 Institute of Pathological Physiology, First Faculty of Medicine, Charles University, 12853 Prague, Czech Republic; 3 Department of Haematology, First Faculty of Medicine, Charles University and General Hospital, 12808 Prague, Czech Republic; jinpol@gmail.com (J.P.); trneny@cesnet.cz (M.T.); 4 Department of Dermatology and Venereology, First Faculty of Medicine, Charles University and General Hospital, 12808 Prague, Czech Republic; 5 Faculty of Mathematics and Physics, Charles University, 18675 Prague, Czech Republic; pesta@karlin.mff.cuni.cz

**Keywords:** cutaneous T-cell lymphomas (CTCL), mycosis fungoides, Sezary syndrome, microRNA, Psoriasis vulgaris, atopic dermatitis

## Abstract

Primary cutaneous T-cell lymphomas (CTCL) affect the skin and tend to transform and spread. CTCL involves primarily the Mycosis fungoides (MF) and more aggressive Sezary syndrome (SS). Oncogenic microRNAs (miRs) are stable epigenetic inhibitors often deregulated in the tumour and detectable as biomarkers in non-cellular fractions of peripheral blood. The tumour-specific expression of miR-155, miR-203, and miR-205 was shown to correctly diagnose CTCL. We herein asked whether these microRNAs can be used as plasma biomarkers for clinical CTCL monitoring. Patients with CTCL (*n* = 10) and controls with non-malignant conditions (*n* = 11) repeatedly donated plasma samples every ca. five months. MicroRNAs were detected in the plasma samples by specifically-primed RT-PCR followed by multivariate analyses of the miR expression dynamics. We herein established the plasma miR-classifier for detecting CTCL based on the miR-155 upregulation and miR-203/miR-205 downregulation with 100% specificity and 94% sensitivity. The 3-miR-score in the consecutive samples coincided with the clinical outcome of MF and SS patients such as the therapy response or changes in the clinical stage or tumor size. Quantitation of the selected microRNAs in plasma is a specific and straightforward approach for evaluating CTCL outcome representing, thus, a valuable tool for CTCL diagnostics and therapy response monitoring.

## 1. Introduction

Primary cutaneous T-cell lymphomas (CTCL) affect the skin in form of lesions that tend to transform and spread into lymph nodes, organs or blood. CTCL is a heterogeneous group of lymphomas that in up to 3/4 of cases present as Mycosis fungoides (MF). Another form of CTCL with overlapping and aggressive features is represented by the Sezary syndrome (SS). Differential diagnosis involves other types of lymphomas or benign lesions (BL) such as Psoriasis vulgaris, atopic dermatitis and other chronic skin diseases all requiring repeated skin histology examinations in order to differentiate the BL from CTCL. Staging systems for MF/SS distinguish surface lesions (stains, plaques, erythrodermia, tumour), as well as lymph node or systemic involvement of the tumour cells [1]. Improving the overall survival and quality of life is among the major goals of current therapeutic interventions of CTCL. Another goal is to evolve sensitive diagnostic and monitoring techniques to distinguish CTCL from benign lesions and to assess therapeutic responses and predict/confirm progression. Such approach may be very beneficial for all CTCL patients, but especially for those with advanced-stage disease potential resulting in nodal, visceral, or blood involvement. Patients in disease progression are treated by biologic-response modifiers or histone deacetylase inhibitors before escalating therapy to chemotherapy or considering an allogeneic stem-cell transplantation. Despite significant effort, the treatment of CTCL does not usually lead to long-lasting complete remission.

CTCL pathogenesis may be a result of chronic antigenic stimulation by viral or bacterial infection resulting into clonal expansion and transformation of T-cells also involving their epigenetic reprograming [2]. There exist predisposing factors to CTCL of viral origin such as EBV, HHV8, HIV, or HCV all previously associated with epigenetic dysregulation of expression programs of noncoding RNAs [3]. Indeed, initially the miR-21 [4] than a complete set of microRNAs (miRs) was identified as pathogenic factor in CTCL [5]. As expected, expression of certain miRs (miR-155, miR-203, miR-205) distinguishes CTCL from benign inflammatory skin disorders with a very high accuracy (95%) implicating great diagnostic potential of miRs [5]. MiRs are small, non-coding RNAs that bind mRNA targets, usually at the 3′ untranslated region, via a set of complementary nucleotides as part of the RNA-induced silencing complex (RISC). MiRs are regulators of gene expression at a post-transcriptional level often targeting multiple mRNAs and at the same time being quite redundant for every mRNA, thus creating a complex epigenetic network of multiple regulatory circuits. Dysregulation of miRs leads ultimately to differences in expression profiles of other miRs as well as their targets.

Work from others, as well as from our group, demonstrated that miRs produced by a tumour (miR-155, miR-24, miR-19a, miR-181b) can be very reproducibly detected in non-cellular fractions of peripheral blood and, thus, can serve as tumour biomarkers [6]. It is also important to note that miRs in serum or plasma are very stable and resistant to RNA degradation, which makes their detection useful for routine clinical monitoring. We herein asked whether detection of miRs previously established for the CTCL tumour tissue is applicable for plasma samples of CTCL patients and whether the miR plasma levels relate to the tumour growth and the therapy-mediated eradication. Our data provide new evidence that monitoring of the peripheral blood miRs may be a very effective method for evaluating CTCL progression as well as determining their distinction from benign lesions (BLs).

## 2. Results

### 2.1. Establishment of the miRNA Classifier for Cutaneous T-Cell Lymphomas (CTCL) Plasma

**Figure 1:**
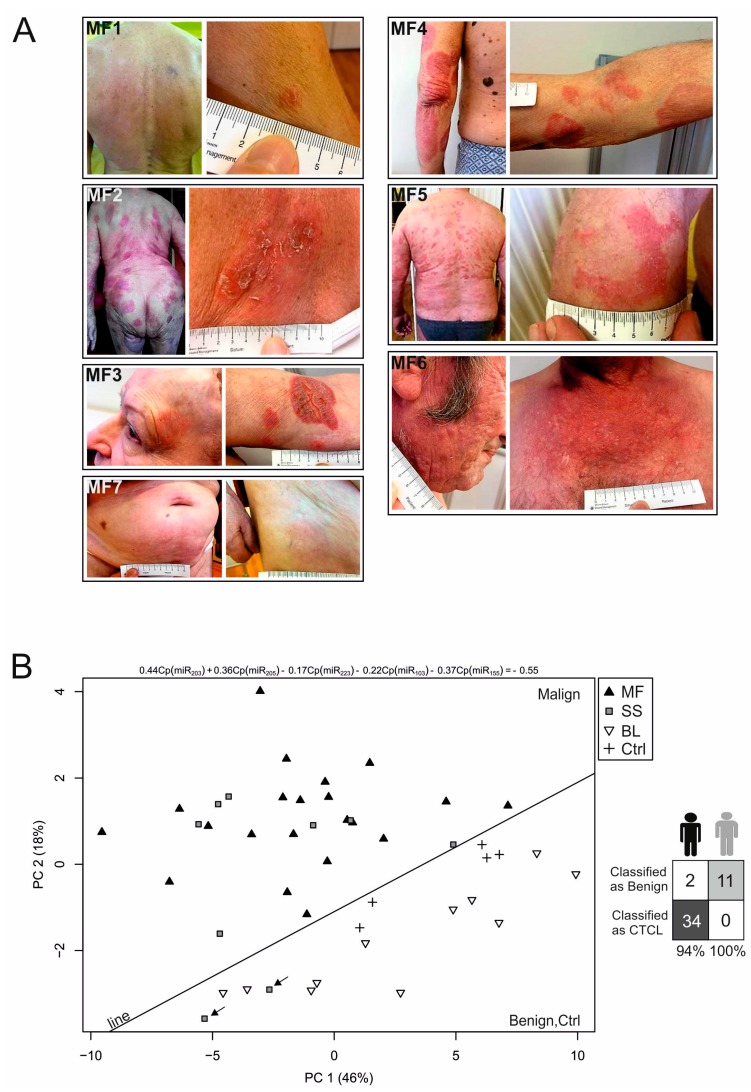
CTCL-MF patients and miR-classifier for CTCL plasma. (**A**) Photographs of the lesions in MF patients (MF1-7). Each panel shows a larger view of the lesion and its detail; (**B**) Principal component analysis (PCA) analysis of the miR-classifier in CTCL, benign lesions (BL) and healthy donors using expression data of miR-155, miR-203, miR-205, miR-22, miR-223, miR-103, and miR423-5p. A line separating the CTCL from BL is defined in the Results section. MF: dark triangle, SS: grey rectangle, BL: empty triangle, and controls are indicated by a cross. The arrows indicate two CR samples of one patient. (**B**) Right: classification performance in the training set using the progressive sampling algorithm called NSC (specificity 100%, sensitivity 94%) in which the dark field represents samples correctly diagnosed as malignant, the grey field represents samples correctly diagnosed as benign, and empty fields represent samples that are falsely diagnosed. Dark person icon: CTCL; grey person icon: BL.

**Figure 2:**
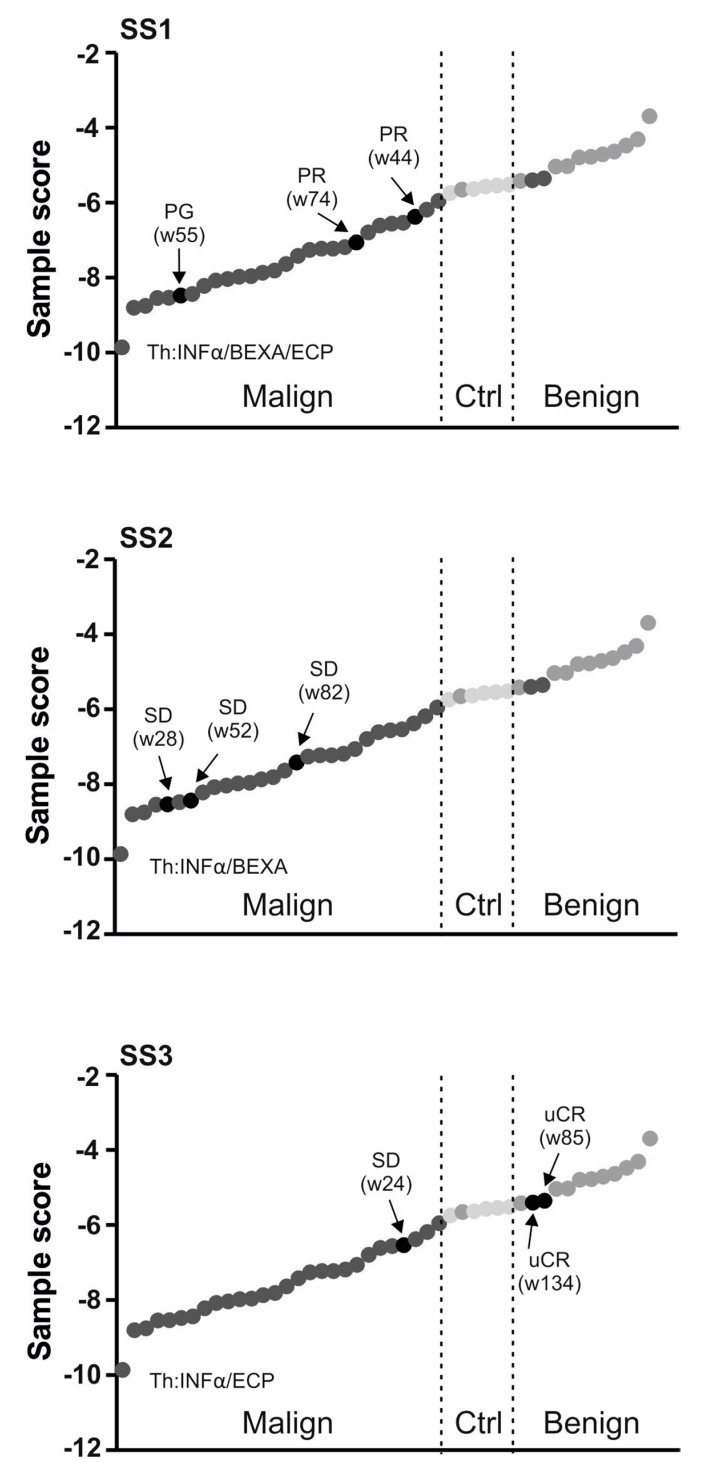
Plasma 3-miR-score in SS patients. Clinical data (SS1-3) are indicated including the therapy (Th) & response (see Table 1). For each sample the time in weeks (w) is calculated from the diagnosis. Each sample represented by a circle: empty healthy, grey BL, dark CTCL. Dashed lines separate diagnostic subgroups. Arrows indicate consecutive samples of a patient whose ID is placed on top.

As pointed above the expression of certain miRs in tumour tissue distinguishes CTCL from BLs with a very high accuracy [5]. It is also established for some cancers that oncomiRs are detectable in non-cellular fractions of peripheral blood. We followed up on this and as a first step determined levels of the miR-155, miR-203, miR-205, miR-223, and miR-22 (together with loading controls: miR-103, miR-423-5p) in CTCL and non-CTCL plasma samples utilizing qPCR. Clinical Table 1 indicates survival, therapy, its response and staging for CTCL patients (see photographs of their lesions, Figure 1A), and also relevant information for controls. To calculate the miR-classifier (a formula for classifying CTCL using microRNA levels) we utilized the Cp values exactly as published previously [5]. Next, we analysed the data with the principal component analysis (PCA) and observed that samples from CTCL became separated from BLs and normal individuals (Figure 1B, left). Interestingly, the PCA analysis indicated that BLs are more distant from CTCL compared to normal individuals, which could be attributed to the fact that the BLs display marked downregulation of miR-155. In addition, very close to the non-CTCL group were two samples indicated by arrows with SS in complete remission (see also Figure 2, patient SS3). Principal components are mutual orthogonal vectors that describe maximal variation in our dataset regarding the miR expression. The first few components explain most of the variance in the dataset (64% variance). Based on the PCA analysis we corroborate a line that separates CTCL from BL samples reasonably well. The formula for that line reads:

(1) 0.44 Cp(miR−203)+0.36Cp(miR−205)−0.17Cp(miR−223)−0.22Cp(miR−103)+0.37Cp(miR−155)=−0.55

indicating the contribution of the studied miR to the data separation. Using the 5-miR classifier, 11 out of 11 patients with BLs were classified as benign, whereas 34 out of 36 CTCL patients were classified as malignant (Figure 1B, right). The specificity of detecting CTCL vs. BL by the miRs in plasma was 100% (confidence interval 71–100%, *p* = 0.001 utilizing exact binomial test) and sensitivity was 94% (confidence interval 81–99%, *p* = 1.941 × 10^−8^).

**Figure 3:**
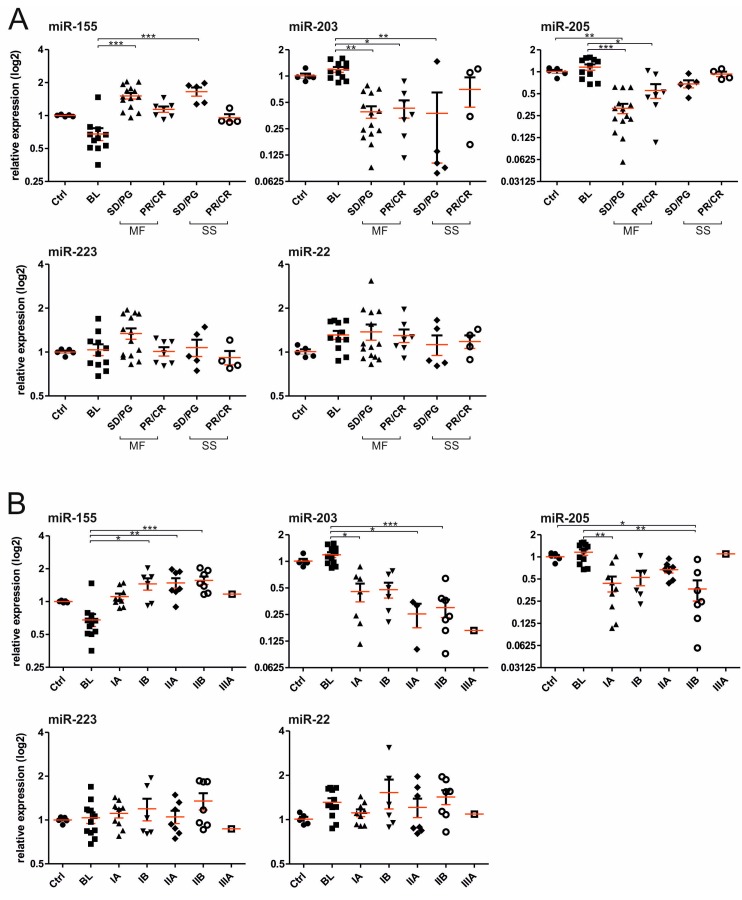
Plasma miR levels and CTCL clinical outcome. miR levels (in log2 scale) shown in respect to (**A**) therapeutic response; and (**B**) clinical stages. Ctrl: healthy control, Abbreviations: BL, MF, SS, SD, CR, PR or PG are described within the text and Table 1. Mean ± SD using the Kruskal-Wallis one-way ANOVA test. * *p* = 0.05, ** *p* = 0.01, *** *p* = 0.001.

To further understand which miRs added the most to the miR-classifier, we display the data of each miR separately in the Figure 3. The data indicate that the miR-155 was significantly upregulated in both MF, as well as SS patients compared to BLs. Correspondingly, levels of miR-203 and miR-205 were significantly downregulated in the CTCL plasma. We also divided the patients’ samples according to the therapy response and observed the trends in reduction of miR-155, as well as the increase of the miR-203 and 205 in patients with the therapy response (partial remission (PR)/CR) for both MF and SS (Figure 3A). The levels of miR-223 and miR-22 were on average not significantly changed compared to controls but the data distribution was slightly more heterogeneous. Next, we show the miR levels (Figure 3B) in respect to clinical stages according to ISCL/EORTC for MF/SS as defined elsewhere [7]. The trends of increasing miR-155 and the reciprocal decreases of miR-203 and miR-205 are observable at higher stages. This is particularly noted between stage I (patches, papules, and plaques) and II (tumour > 1 cm, or lymph node involvement). Again, no trends were noted for miR-223 and miR-22 and staging. Therefore, the miR-classifier separated patients with CTCL compared to controls and BLs and further indicated that patients with increased tumour burden have higher levels of miR-155, and lower levels of miR-203 and miR-205. Upon responding to the therapy, the levels of miR-155, miR-203, and miR-205 tend to normalize, which became the subject of our next analyses.

### 2.2. Use of miRNA Classifier for the CTCL Clinical Monitoring

**Figure 4:**
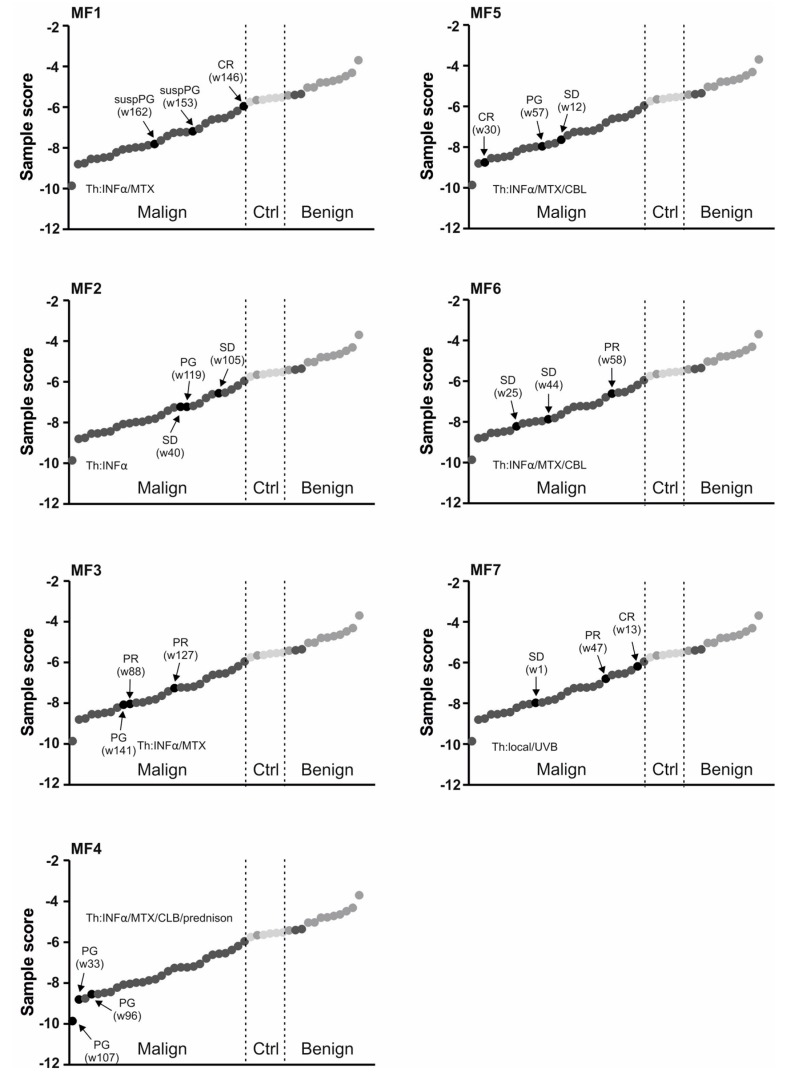
Plasma 3-miR-score in MF patients. Clinical data (MF1-7) are indicated, including the therapy (Th) and response (see Table 1). For each sample the time in weeks (w) is calculated from the diagnosis. Each sample represented by a circle: empty healthy, grey BL, dark CTCL. Dashed lines separate diagnostic subgroups. Arrows indicate consecutive samples of a patient whose ID is placed on top.

To further study the role of the miR classifier utilizing the most influential miRs (miR-155, miR-203, and miR-205), we asked how it reflects the clinical context of CTCL. As indicated already by PCA analyses some samples tend to stay closer to the BLs and we noted that these samples were those obtained from the clinically-responding patients (CR). Therefore, we next analysed the 3-miR-score of MF patients in three consecutive samples in respect to the clinical outcome (Figure 4). The patients that achieved either CR or PR markedly increased the value of the 3-miR-score to the level found in controls/BLs. Examples include the patients MF1, MF6, and MF7. Conversely, upon disease progression (PG) in four MF patients (MF1, MF2, MF3, MF4) the level of the 3-miR-score markedly decreased. Figure 2 shows results of the SS patients again analysed in the three consecutive samples. The two SS patients (SS1, SS3) increased the value of the 3-miR-score upon achieving PR/CR while, in turn, the progression (seen in the patient SS1) was characterized by a marked decrease of the 3-miR-score. Taken together, the 3-miR-score used for plasma samples of the CTCL patients may become very valuable tool for analysing the CTCL patients’ therapy response.

**Figure 5:**
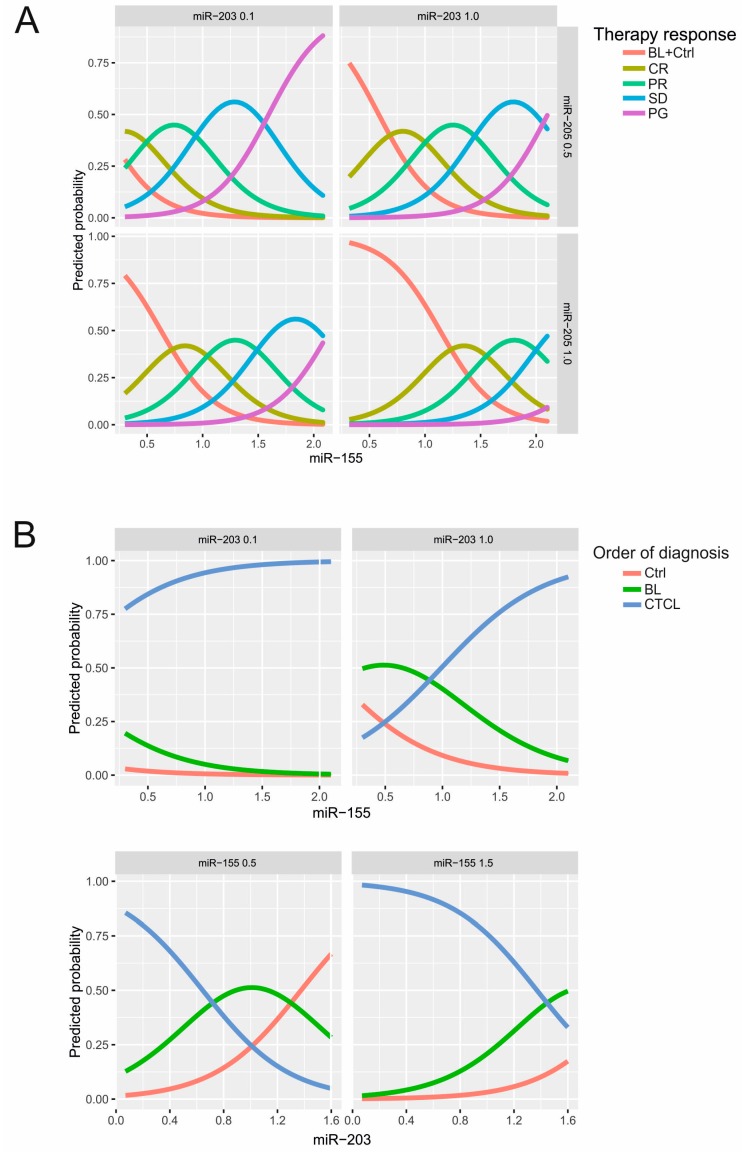
Predicted probability of therapy response and diagnosis based on miR levels. (**A**) Predicted probability that a patient has a particular therapy response (CR, PR, SD, PG) or belongs to the BL/healthy control. The *X*-axis provides miR-155 level at certain levels of either miR-203 (0.1 vs. 1.0) or miR-205 (0.5 vs. 1.0); (**B**) predicted probability that a patient is healthy, benign, or malignant (Ctrl < BL < CTCL, i.e., ordered diagnosis) based on the ordered logistic regression model. Increasing miR-155 (*X*-axis) increase the probability of belonging to the CTCL category seen at two levels of miR-203. Bottom panel: accordingly, decreasing miR-203 (*X*-axis) increase the probability of belonging to the CTCL category seen at two levels of miR-155 (0.5 vs. 1.5).

We next evaluated the following orders of benefit from therapy using the multivariate ordinal logistic regression analysis in CTCL patients. These orders included the therapy response (from complete remission CR to partial remission PR, stable disease SD, and finally to the progression PG; CR < PR < SD < PG), clinical stages (IA < IB < IIA < IIB < IIIA), and tumour size (skin: 1 < 2 < 3 < 4). Values of miRs analysed in plasma of the control (Ctrl), BLs, and CTCL also represented such order of diagnoses (Ctrl < BL < CTCL). Results summarized in the Table 2 indicate that miR-155 and miR-203 significantly influence belonging to any of the four orders, while miR-205 influences only the therapy response. The other miRs (miR-223, miR-22) or other routinely determined parameters in the plasma (LDH, β2-microglobulin) had no effect on the belonging to any of the orders of benefit. Graphically the data are also presented in the Figure 5A where higher miR-155 levels and lower miR-203 as well as miR-205 levels increase the probability of belonging to non-response’s category. To conclude, while the miR-155 increase suggests a higher probability of disease progression, the same is predicted by a lower level of miR-203 or miR-205 (Figure 5, Figures S1 and S2).

## 3. Discussion

miRs are very stable epigenetic modifiers of posttranscriptional expression profiles, often involved in tumourigenesis. miR stability precludes its use as the disease biomarker. We herein evaluated the expression of candidate miR-oncomarkers in CTCL in the plasma of patients with MF and SS in several disease time-points, which allowed us to compare how dynamic changes in miR level relate to CTCL biology and clinical outcomes. miR-155, miR-203, and miR-205 were indeed differentially regulated in CTCL compared to control samples from patients with BLs or normal individuals. In addition, the predictive 3-miR-score previously generated for tumour tissues [5] and validated on independent patient cohort [8] successfully separated the CTCL from controls and BLs when implementing the data obtained from plasma (Figure 1 and Figure 3). The size of the separation effect by PCA was, however, relatively smaller compared to data generated from larger microarray datasets [5]. The studied miRs are very unique in CTCL biology as previously suggested by another study [9]. While miR-155 acts as an oncogene in many different tumour cell types, the miR-203 and miR-205 are putative tumour suppressors expressed in keratinocytes. Another work implicates that downregulation of miR-203 and miR-205 perturbs epidermal differentiation [10] and accelerates cell proliferation [11]. In turn, miR-155 is epigenetically activated via oncogene MYB that binds and activates chromatin structure near the miR-155 host gene [12]. As a result, the miR-155 upregulation blocks normal immune function, as well as staying behind the handful of lymphoid malignancies [13] by targeting important transcription and signalling factors such as PU.1 or SHIP1-PIP3-AKT pathways. Another study indicated that the STAT5/BIC/miR-155 pathway may be responsible for controlling cell proliferation during malignant T-cell transformation [14]. This notion is also supported by a mouse model with miR-155 overproduction [15] that results in lymphomas. Interestingly, both malignant, as well as occasionally non-malignant T-cells within the CTCL/MF tumour biopsies stained positive for miR-155 using an in situ hybridization technique [16]. The three miRs may potentially mediate mechanisms responsible for hijacking the inflammatory responses by malignant transformation as reviewed elsewhere [17]. Thus, the oncogenic and tumour suppressive miRs guiding the immune homeostasis may become epigenetically dysregulated as a result of transformation to CTCL.

It is very important to correctly diagnose CTCL from benign lesions, as well as to establish sensitive and specific CTCL monitoring of potential relapse. Our work suggests that this can be achieved via specific microRNA detection in plasma. As shown in Figure 2 and Figure 4 the clinical outcomes were often presumed by changes in the miR score. Indeed, the levels of the three most important miRs (miR-155, miR-203, miR-205) had significant effects on the therapy response, while miR-155 and miR-203 had additionally significant effects on belonging to a clinical stage and on the tumour size (Figure 5). The advantage of our work compared to previous data from skin biopsies [5] is that it allows utilization of peripheral blood plasma, which is relatively easy to obtain at routine patient visits and can be obtained periodically. As a result, the miR data are quantitative and highly reproducible. Significance in the predictive score is partly due to the fact that miR-155 is directly involved in the biology of MF progression [9] as also supported elsewhere [18]. Interestingly, the upregulation of miR-155 alone is not able to completely distinguish between early MF and inflammatory dermatoses and to separate patients and controls, as well as tumours from the intraepithelial lymphocytes [18], further supporting the use of the multi-miR-classifier. The score, in fact, contains another two miRs, miR-223 and miR-22, which were previously shown upregulated in the CTCL tumour tissue [5] while in the MF or SS plasma (as shown in the Figure 3) the levels of miR-223 and miR-22 were not significantly affected. Moreover, as shown by another study, levels of miR-223 are reduced in CTCL while their targets became reciprocally upregulated [19]. Similarly, another study noted a reduction in miR-223 during progression of MF to advanced MF [19]. In addition, reduction of miR-22 was noted in both CTCL forms, MF and SS, compared to controls by another study [20].

Taken together, the tool involving the miR detection in plasma allows both to correctly diagnose CTCL from benign lesions as well as to establish sensitive and specific monitoring of CTCL progression. Although our work represents initial findings on relatively small patient cohort we are confident that associating the miR levels with clinical outcome allows to initiate further validation studies in the CTCL patients.

## 4. Material and Methods

### 4.1. Patients

Patients with CTCL (MF, SS) donated three plasma samples with a median interval between samples of five months (2–14 Mo.). The diagnosis of CTCL was established according to the WHO2008 criteria. MF patients (*n* = 7) of median age was 66 years (61–83) and three SS patients (71, 61–72 years) as well as the controls of either five healthy volunteers (52, 44–65 years) or six BL patients with Psoriasis vulgaris or atopic dermatitis (56, 31–85 years) participated in the study. Samples were collected between 2012 and 2016 in collaboration between the Haematology and Dermatology clinical departments of General Hospital Prague. All samples were obtained upon written informed consent according to the Helsinki Declaration and approved by the internal ethics committee (#43/13, 29.10.2013). Patients’ characteristics are shown in the Table 1. To document the extent of lesions in MF, the photographs are provided (Figure 1A) with permission from each patient.

### 4.2. MiR Extraction and Quantitation

RNA was isolated using an miRNeasy^®^ Mini Kit (Qiagen, Hilden, Germany) from 200 μL of plasma (dissolved in 1 mL of QIAzol^®^ Lysis reagent (Qiagen, Hilden, Germany)) and processed with several modifications exactly as published previously [6]. Reverse transcription was performed using a High Capacity cDNA Reverse Transcription Kit supplemented with miR-specific primers (Thermo Fisher Scientific, Waltham, MA, USA). The amplification was performed by the LightCycler Version 1.5.0.SP3 software 480 real-time PCR system (Roche, Basel, Switzerland) in 384-well plates. Quantitative polymerase chain reaction was run for 45 cycles of 95 °C for 15 s and 60 °C for 1 min. Relative expression was calculated from Cps of the oncomiR (miR-155, miR-203, miR-205, miR-223, miR-22) relative to previously-established loading controls of miR-103 and miR-423-5p in CTCL vs. healthy plasma using the 2^−(ΔCp)^ equation in duplicate samples [5]. PCA was used to further analyse the data. PCA uses orthogonal transformation to reduce the number of dimensions given by seven expression Cp values of miRs and converts the datasets into the space of principal components. The equation to calculate the 3-miR-classifier using Cp values of miR-155, miR-203, and miR-205 is:

(2) $$S=\mathrm{Cp}\;\mathrm{mir}155-\mathrm{Cp}\frac{\mathrm{mir}203}{2}-\mathrm{Cp}\frac{\mathrm{mir}205}{2}$$

### 4.3. Statistical Analysis

MiR expression data were evaluated using a multivariate proportional odds regression model together with the likelihood ratio test to associate their levels to belonging to one of the four orders of benefit (therapy response, clinical stage, tumour size, and diagnosis). The applied analysis is appropriate as the model takes into account the ordinary character of the categorical variables.

## 5. Conclusions

The determination of plasma levels of oncogenic microRNA miR-155 and two tumour-suppressor microRNAs, miR-203 and miR-205, represents a very useful tool for both making diagnostic distinctions from benign skin lesions, as well as for monitoring of CTCL patients utilizing peripheral blood plasma samples. Furthermore, this 3-miR-score relates to CTCL clinical responses and in the near future may be used when introducing novel therapies to evaluate their efficacy and assessing the remaining tumour burden.

## Figures and Tables

**Table 1:** Clinical data of the T-cell lymphomas (CTCL) patient cohort.

ID	Age	Sex	DG	Stage S1	OS	Alive	Therapy	Response
MF1	61	M	MF	IB	58	Y	IFNα/LD MTX	CR
MF2	74	F	MF	IB	92	Y	IFNα	SD
MF3	66	F	MF	IB	179	Y	IFNα/LD MTX	PR
MF4	82	M	MF	IIB	68	Y	IFNα/CLB/MTX/PRD	SD
MF5	62	M	MF	IB	28	Y	IFNα/LD MTX/CLB	CR
MF6	61	M	MF	IIB	57	Y	IFNα/LD MTX/CLB	PR
MF7	70	F	MF	IA	23	Y	local Th/UVB	CR
SS1	72	F	SS	IIIA	94	Y	IFNα/BEXA/ECP	PR
SS2	71	F	SS	IIA	40	Y	IFNα/BEXA	SD
SS3	60	M	SS	IIA	44	N	IFNα/ECP	uCR

MF: Mycosis fungoides, SS: Sezary syndrome, IFNα: interferon alpha, LD MTX: low dose methotrexate, CLB: chlorambucil, MTX: methotrexate, PRD: prednisone, ECP: extracorporeal photopheresis, BEXA: Bexarotene, UVB: phototherapy, PR/CR: partial or complete remission, uCR: uncertain CR, SD: stable disease, OS: overall survival (in months) calculated from the diagnosis. Stage S1: clinical stage of Sample 1.

**Table 2:** Effect of miR-levels on probability of the CTCL progression.

Clinical Parameter	miR-155	miR-203	miR-205	miR-223	miR-22
Th. Response	0.00022	0.01758	0.00070	0.12323	0.08071
Clinical Stage	0.00404	0.00029	0.5580	0.5714	0.4340
T (skin)	0.0042	0.0004	0.7792	0.2813	0.8939
Diagnosis	0.0332	0.0034	0.234	0.684	0.071

Legend: *p*-values determined using the likelihood ratio test from the multivariate ordered logistic regression between miR levels and four different orders of benefit.

## Supplementary Materials

The following are available online at www.mdpi.com/1422-0067/18/10/2136/s1.

## Abbreviations

miR	microRNA
CTCL	Cutaneous T-cell lymphomas
MF	Mycosis fungoides
SS	Sezary syndrome
BL	Benign lesion
PCA	Principal component analysis
Cp	Crossing point
CR	Complete Remission
PR	Partial Remission
SD	Stable Disease
PG	Progression
