# Supplementary material for: Plasma miR-155, miR-203, and miR-205 are Biomarkers for Monitoring of Primary Cutaneous T-Cell Lymphomas
Source: Int J Mol Sci. 2017 Oct 15;18(10):2136. doi: 10.3390/ijms18102136 (PMC5666818; doi:10.3390/ijms18102136)
Supplement: Supplementary file 1 [file ijms-18-02136-s001.pdf]

Plasma miR-155, miR-203, and miR-205 are Biomarkers for Monitoring of Primary Cutaneous T-cell Lymphomas

Nina Dusílková [1,2,+], Petra Bašová [1,+], Jindřich Polívka [3], Ondřej Kodet [1,4], Vojtěch Kulvait [1], Michal Pešta [5], Marek Trněný [3] and Tomáš Stopka [1,3,*]

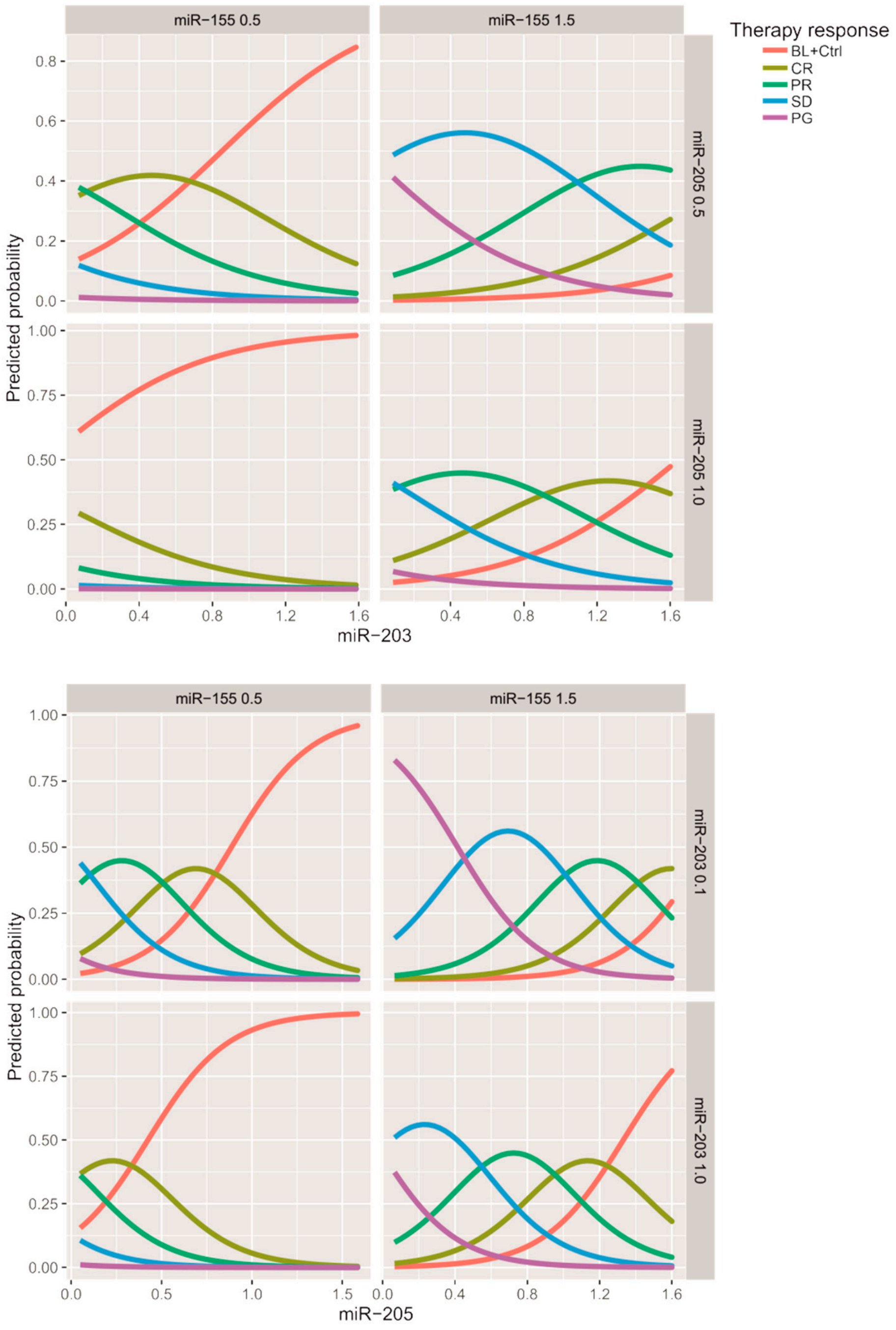

Figure S1. Predicted probability of therapy response based on miR levels. Predicted probability that a patient has a particular therapy response (CR, PR, SD, PG) or belong to BL/healthy control. Upper 4 panels: Influence of miR-203 level (X axis) at certain levels of either miR-155 (0.5 vs 1.5) or miR-205 (0.5 vs 1.0). Lower 4 panels: Influence of miR-205 level (X axis) at certain levels of either miR-155 (0.5 vs 1.5) or miR-203 (0.1 vs 1.0).

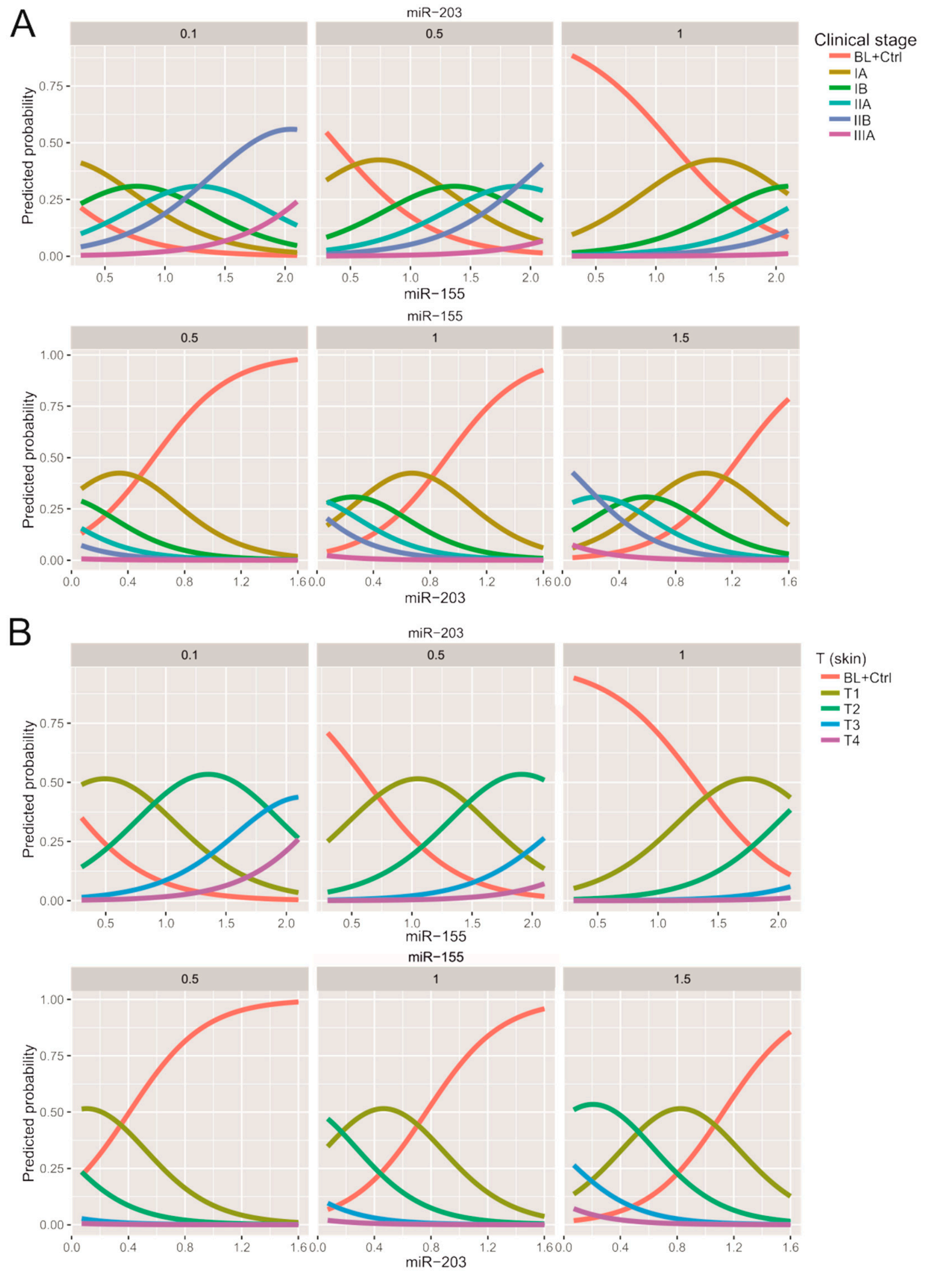

Figure S2. Predicted probability of belonging to particular diagnosis or tumor size based on miR levels. (**A**) Predicted probability that a patient is healthy, benign or at 5 malignant stages (IA, IB, IIA, IIB, IIIA) based on the ordered logistic regression model. Increasing miR-155 (X axis) increase the probability of belonging to the progressed stage of CTCL seen at three levels of miR-203 (0.1, 0.5, 1.0). Bottom panel: accordingly, decreasing miR-203 (X axis) increase the probability of belonging to the progressed stage of CTCL seen at three levels of miR-155 (0.5, 1.0, 1.5); (**B**) Predicted probability that a patient is healthy, benign or at 4 malignant tumor stages (T1-T4) based on the ordered logistic regression model. Increasing miR-155 (X axis) increase the probability of belonging to the larger tumor size of CTCL seen at three levels of miR-203 (0.1, 0.5, 1.0). Bottom panel: accordingly, decreasing miR-203 (X axis) increase the probability of belonging to the larger tumor size of CTCL seen at three levels of miR-155 (0.5, 1.0, 1.5).
